# Attenuation of Chikungunya Virus by a Single Amino Acid Substitution in the nsP1 Component of a Non-Structural Polyprotein

**DOI:** 10.3390/v17020281

**Published:** 2025-02-18

**Authors:** John Chamberlain, Stuart D. Dowall, Jack Smith, Geoff Pearson, Victoria Graham, John Raynes, Roger Hewson

**Affiliations:** 1 UK Health Security Agency (UK), Porton Down, Salisbury SP4 0JG, UK; 2 Faculty Infectious and Tropical Diseases, London School of Hygiene and Tropical Medicine, Keppel Street, London WC1E 7HT, UK

**Keywords:** Chikungunya virus, Togaviridae, mutation, attenuation, A533V

## Abstract

Chikungunya virus (CHIKV) is a mosquito-transmitted alphavirus that, since its re-emergence in 2004, has become recognised as a major public health concern throughout many tropical and sub-tropical regions of the world. Amongst the insights gained from studies on other alphaviruses, several key determinants of virulence have been identified, including one present at the P3 position in the nsP1/nsP2 cleavage domain of the S.A.AR86 Sindbis (SINV) strain. This strain is associated with neurovirulence in adult mice; however, when a threonine-to-isoleucine substitution is engineered at this P3 position, an attenuated phenotype results. A reverse genetics system was developed to evaluate the phenotype that resulted from the substitution of alanine, present at the P3 position in the wild-type CHIKV clone, with valine. The A533V-mutant CHIKV induced milder disease symptoms in the C57BL/6 mouse model than the wild-type virus, in terms of severity of inflammation, length of viraemic period, and histological changes. Furthermore, the induction of type I IFN occurred more rapidly in both CHIKV-infected cell cultures and the mouse model with the mutant CHIKV.

## 1. Introduction

Chikungunya virus (CHIKV), the aetiological agent of chikungunya fever, is classified in the *Alphavirus* genus within the family *Togaviridae*. Three major CHIKV genotypes are recognised, each named in accordance with the region in which the strain was first isolated: Asian, West African, and East Central South African (ECSA). Transmission to humans occurs predominantly through the bite of virus-infected *Aedes aegyptii* or *A. albopictus* mosquitos [1,2]; however, mother-to-child transmission (both intrapartum and maternal–foetal) have also been reported [3].

Seroprevalence studies following CHIKV outbreaks indicate that a relatively low proportion of infected individuals (3–15%) remain asymptomatic [4,5], when compared to other Old World alphaviruses, such as Sindbis virus (SINV) and Ross River virus (RRV) [6,7]. Symptoms develop following an incubation period of approximately 3–7 days and are typically characterised by the abrupt onset of a high fever (>39 °C), erythematous, maculopapular rash, and severe poly-arthralgia [4,5,8]. Whilst the majority of cases resolve in 7–14 days, some patients experience persistent poly-arthralgia or arthritis lasting for many weeks or months. Other common symptoms include headache, photophobia, fatigue, vomiting, diarrhoea, myalgia, and conjunctivitis. A small proportion of chikungunya patients, largely comprising those with underlying medical conditions, young children, and the elderly, may develop any of a range of severe complications, including, myocarditis, hepatitis, neurological disorders, and multi-organ failure [3].

Where the alleviation of symptoms occurs in acute CHIKV infections, it follows soon after the induction of the innate immune response and prior to the appearance of protective antiviral antibodies [9,10,11]. Evidence of the crucial role played by type I IFN in controlling infection has been demonstrated in both human cases and mouse models, which show that in its absence, viral dissemination and disease severity are increased [10,11,12,13].

Alphavirus virions consist of spherical particles with a diameter in the range of 60–70 nm, enclosed in a lipid envelope derived from the host cell plasma membrane, which contains the virus-encoded glycoproteins E1 and E2, forming heterodimers that are grouped as trimers to form 80 spikes on the virion surface [1,2]. Within the envelope, a viral capsid shell encloses a linear single-stranded, positive-sense RNA genome of approximately 11–12 Kb. The genome is flanked at the 5′ end by a 7-methylguanosine cap and at the 3′ end by a poly-A tail and contains two open reading frames (ORFs). The ORFs encode the non-structural or replicase polyprotein and the structural polyprotein, expressed via a sub-genomic RNA. The polyproteins are processed to produce four non-structural proteins (nsP1, nsP2, nsP3, and nsP4) and five structural proteins (C, E3, E2, 6K, and E1).

The cleavage of the non-structural (ns) polyprotein (nsP1234) occurs in a highly regulated sequence to four final products, mediated through the protease activity of a domain located near the C-terminal of nsP2. This stepwise process results in the presence of intermediate cleavage products prior to the formation of the mature replicase complex. Each of these favours the synthesis of specific viral RNA species (full-length negative-sense RNA, genomic RNA, and sub-genomic RNA), which act to regulate virus replication [1,14,15]. A range of functions associated with nsPs have been determined: nsP1 is the viral capping enzyme and membrane anchor of the replication complex, while nsP2 is an RNA helicase and the protease responsible for ns polyprotein processing. NsP3 interacts with several host proteins and may modulate protein poly- and mono-ADP-ribosylation, and nsP4 is the core viral RNA-dependent RNA polymerase.

In common with members of other virus families, alphaviruses have evolved mechanisms by which they can evade or block the host antiviral IFN response both by invoking a general shutoff of host gene expression and by targeting specific components of the IFN signalling pathways, thereby contributing to pathogenesis. Several key virus-encoded motifs instrumental in mediating these antagonistic effects have been identified in both structural and non-structural viral proteins and as such represent potential targets for the development of novel antiviral agents [16,17,18,19,20,21]. However, determinants targeting a particular component of the host immune system may not be located in the same protein in all members of the genus. This is exemplified by the differing mechanisms employed by alphaviruses to promote host cell transcriptional shutoff; in the Old World alphaviruses SINV, Semliki Forest virus (SFV), and CHIKV, this process is associated with a determinant located in nsP2, whereas in the New World alphaviruses, Venezuelan equine encephalitis (VEE) and Western equine encephalitis (WEE), a component of the capsid protein is required [18].

A determinant present in the P3 position of the nsP1/nsP2 cleavage site of the non-structural polyprotein of the SINV strain S.A.AR86 has been shown to be critical for inducing adult mouse neurovirulence [16] and the introduction of a single amino acid substitution at this site (T538I) attenuates this phenotype. It was subsequently shown that the introduced mutation disrupts a mechanism whereby type 1 IFN induction is inhibited in the host cell by the wild-type virus [22]. Furthermore, the authors reported a similar role for the amino acid present at the analogous site in Ross River virus (RRV).

In this study, infectious cDNA clones were constructed from a CHIKV isolate belonging to the ECSA genotype in order to investigate the analogous region in this species. CHIKV in common with the majority of alphaviruses contains alanine at the P3 position [1]. The wild-type clone containing alanine and a different clone in which a valine substitution had been engineered, were evaluated. An investigation into the resulting phenotypes both in vitro and in vivo, indicated that a similar determinant is present in CHIKV, and that it also plays a role in inhibiting the host type 1 interferon response.

## 2. Materials and Methods

### 2.1. Cell Culture and Virus Stocks

BHK-21 (baby hamster kidney fibroblasts), Vero (adult African Green monkey epithelial cells), and L929 (mouse fibroblasts) cell lines were obtained from the European Collection of Authenticated Cell Cultures (ECACC), Porton Down, Salisbury, UK. (ECACC catalogue numbers 85011433, 84113001, and 85011425, respectively). Cells were maintained in Dulbecco’s Modified Eagle’s Medium (DMEM) supplemented with 5% foetal bovine serum (FBS) were in all cases incubated at 37 °C in an atmosphere of 5% CO_2_ and 6% humidity.

The virus strain used in this study, SL-R233 (2006), was isolated in Vero cells from one of a panel of blood serum specimens taken from febrile patients in Columbo, Sri Lanka, between 2006 and 2007 and supplied by Dr Mark Bailey (Heartlands Hospital, Birmingham, UK). A working stock prepared from a second passage in Vero cells was used as the wild-type virus in the remainder of this study.

### 2.2. Nucleotide Sequence Determination

Viral RNA was extracted from the supernatant of CHIKV-infected Vero cell cultures using the QIAamp^®^ Viral RNA Mini Kit. The genome sequence was determined using an Applied Biosystems 3730 DNA analyser from overlapping cDNA fragments amplified by RT-PCR using a Superscript III One-step RT-PCR Platinum^®^ Taq HiFi kit (ThermoFisher Scientific, Dartford, UK). Manipulation and analysis of the nucleotide sequence data obtained were undertaken using the Lasergene suite of programs (DNAStar, version 17.2). The final genomic sequence was deposited in GenBank under the accession number MK086029. Block-based PCR and RT-PCR assays were performed using an Applied Biosystems 2720 Thermal cycler. Reactions were performed at the following conditions: 50 °C for 30 min, 94 °C for 2 min, 40 cycles of (1) 94 °C for 15 s, (2) 55 °C for 30 s, and (3) 68 °C for 1 min per Kb of anticipated amplicon, and finally a single cycle of 68 °C for 5 min. Products separated by agarose gel electrophoresis were visualised by ethidium bromide staining followed by exposure to UV light.

### 2.3. Construction of a CHIKV cDNA Clone

**Figure 1:**
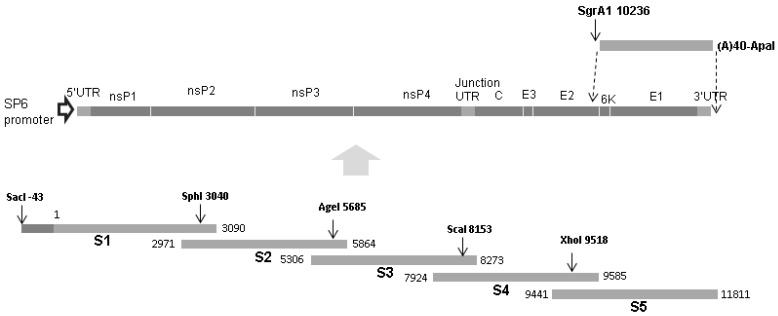
Construction of a full-length clone of CHIKV: Overlapping amplicons representing the entire CHIKV genome (A1-5) were constructed by RT-PCR and combined with the Zero Blunt^®^ TOPO^®^ plasmid to produce sub-clones S1–S5 (A). The genomic fragments were combined in the pGEM^®^ 5Zf(+) vector between the SacI and ApaI restriction sites downstream from the SP6 RNA polymerase promoter. In addition to utilizing unique restriction sites (SphI, AgeI, ScaI, and XhoI) naturally present in the CHIKV genome, the SacI and ApaI sites present in the multiple cloning sites in sub-clones S1 and S5 were used. Two further PCR steps were used to construct a cassette comprising the 3′ terminal 1575 nucleotides of the CHIKV genome followed by a stretch of 40 adenosines and an ApaI site. This product was used to replace the analogous region in the clone.

A full-size clone was constructed from five overlapping sub-genomic amplicons that covered the entire genome (primers detailed in Table 1 and Table 2). These were extracted using the Qiagen QIAquick^®^ Gel Extraction kit and used to construct sub-genomic clones using the Zero Blunt^®^ TOPO^®^ PCR Cloning kit (ThermoFisher Scientific). The virus-specific inserts were subsequently reassembled to make a full-length wild-type clone pCHIK-SL(-) within a pGEM^®^ 5Zf(+) backbone (Promega, Southampton, UK), by utilising restriction sites in the overlapping regions (Figure 1).

A 3′ stretch of 40 adenosines followed by an ApaI restriction site was added to the 3′UTR in two incremental PCR steps. First, an amplicon of 1575 bp was produced using the primers CH9384F and CHIK3′(A15)R with pCHIK-SL(-) as the template, and next the amplicon was amplified using primers CH9384F and CHIKV3′(A40)ApaI (Figure 1). The poly-(dA) tailed product was digested with SgrAI and ApaI and used to construct a chimera with the analogous 3′ region in the full-length wild-type cDNA clone, pCHIK-SL(wt). –A second clone, pCHIK-SL(A533V), was produced from pCHIK-SL(wt) by introducing an alanine to valine substitution at the P4 position in the nsP1/nsP2 cleavage site by site-directed mutagenesis using the Stratagene QuikChange^®^II Site-Directed Mutagenesis kit.

### 2.4. In Vitro Transcription

Plasmid DNA linearized by digestion with ApaI was used as the template for in vitro transcription. Capped RNA transcripts were synthesised by utilizing the SP6 polymerase promoter situated upstream from the CHIKV insert using the mMESSAGE mMACHINE^®^ SP6 transcription kit according to the manufacturer’s instructions (Ambion™ ThermoFisher Scientific, Dartmouth, UK). RNA transcripts were purified using the MEGAclear™ kit (Ambion™ ThermoFisher Scientific, Dartmouth, UK), concentrated by ethanol precipitation and resuspended in 20 µL nuclease-free water.

### 2.5. In Vitro Transfection

BHK-21 cells were transfected with aliquots of a purified CHIKV RNA transcript by electroporation (a single pulse of 140 kV at a 25 F capacitance) using the Genepulser Xcell™ (BioRad, Watford, UK) followed by culture in DMEM.

### 2.6. CHIKV Rescue

Supernatants containing rescued wild-type and A533V-mutant virus were harvested following a single freeze–thaw cycle in which infected cells were frozen at −80 °C for a minimum of 60 min and thawed at room temperature. Supernatant was separated from cellular debris by centrifugation (1000× *g* for 10 min) and stored in 1 mL aliquots at −80 °C. The genomic sequence of rescued virus was determined from extracted RNA.

### 2.7. Virus Titration

Plaque assays were performed on supernatants in triplicate using 6-well assay plates seeded with Vero cells grown to approximately 90% confluence. Following the removal of the culture medium, the cell monolayers were washed with Dulbecco’s phosphate buffered saline (DPBS) and infected with 250 µL of 10-fold virus dilutions prepared in DPBS and incubated for 60 min under standard cell culture conditions as described above. The excess virus was removed from each well and replaced with 3 mL of molten overlay consisting of 0.5× DMEM supplemented with 5% FBS and 1% low-gelling-temperature (LGT) agarose (Sigma-Aldrich A9045, Poole, UK) equilibrated to 40 °C. The overlay was left to solidify at room temperature, then incubated for 72 h. The monolayer was fixed by adding 2 mL of 4% formaldehyde solution in PBS and incubating at room temperature for 60 min. Monolayers were stained by incubating at room temperature for a minimum of 30 min with 0.2% crystal violet diluted in 50% methanol. The excess stain was removed and the cells rinsed with water. After air-drying, plaques were measured and counted, and the titres were estimated.

### 2.8. Analysis of Virus Growth Kinetics

For single-step growth curves, a series of 25 cm^2^ flasks containing Vero or L929 cells were infected with plaque-purified stocks of wild-type or A533V-mutant virus at a multiplicity of infection (MOI) of 0.01. Flasks were incubated for a range of time periods post-infection prior to the harvesting of supernatants and titration by plaque assay.

### 2.9. Statistical Analysis

Minitab software version 16 (Minitab Inc., Coventry, UK) was used to conduct the non-parametric Mann–Whitney statistical test for the statistical analysis of all treatment and control groups. A value of *p* < 0.05 was considered to be significant.

### 2.10. Mouse Infection and Disease Monitoring

To investigate the effect of the A533V mutation on the virus phenotype in vivo, this study was conducted as a pilot, using the mouse model described by Gardner et al. (2010) [23]. Adult female C57BL/6 mice (aged at least 6 weeks old) were purchased from Harlan Laboratories, UK. All animals were treated in strict accordance with the UK Animals (Scientific Procedures) Act 1986.

Three groups of 18 mice were inoculated subcutaneously into the ventral side of both hind feet with 40 µL of inoculum consisting of culture medium containing either 1 × 10^4^ pfu wild-type virus, 1 × 10^4^ pfu A533V-mutant virus, or culture medium only (groups 1, 2, and 3, respectively). An additional group consisting of 3 mice (group 4) received no treatment. Three mice from groups 1–3 were humanely culled at each of six pre-defined time periods (days 1, 3, 6, 9, 12, and 15 post-challenge) by the administration of an anaesthetic overdose. The group 4 mice were culled on day 9.

Visual clinical assessments were conducted twice daily in order to identify symptoms such as postural changes, lethargy, or ruffled fur that might indicate the onset of disease. Prior to the study period, a microchip implant (*idENTICHIP* with Bio-Thermo™, Animalcare Ltd., York, UK) facilitating identification and body temperature monitoring was inserted into the subcutaneous tissue of the neck region of each mouse according to the manufacturer’s instructions. Body temperature, weight, and degree of foot swelling in each animal were monitored on a daily basis. The latter was assessed by determining the height and width of the perimetatarsal area of each hind foot using digital callipers.

### 2.11. Histopathological Investigations

The following tissues were collected post mortem for analysis: both hind limbs, axillary lymph nodes, inguinal lymph nodes, spleen, and liver. The left limb from each animal was placed in 10% neutral buffered formalin for histopathological examination. The right limb, lymph nodes, spleen, and liver were stored at −80 °C prior to processing for molecular studies. The whole limb was decalcified using 10% formic acid and processed to paraffin wax; 5–6 µm sections were cut and stained with haematoxylin and eosin (H&E). Sections were examined by light microscopy; these were read “blind” by a veterinary pathologist in order to avoid bias. The extent of pathological change was subjectively assessed as minimal, mild, moderate, or marked.

### 2.12. Tissue Analysis by qRT-PCR

Tissue samples were weighed, suspended in 1 mL chilled DPBS, and homogenized using a Precellys^®^ 24 tissue homogenizer (Bertin Technologies, Basingstoke, UK). Samples were subjected to 3 cycles of 6200 rpm for 5 s separated by 30 s pauses. Template RNA was extracted from homogenised tissue supernatant using the Qiagen QIAamp^®^ Viral RNA Mini Kit. The determination of viral load and host gene expression was achieved by qRT-PCR using the Applied Biosystems 7500 Fast Real-Time PCR System. The average cycle threshold (Ct) values were normalized against those of the endogenous reference gene hypoxanthine guanine phosphoribosyl transferase (HTRP) using the Qiagen Rn_Hprt1_QF_1 QuantiFast^®^ Probe qRT-PCR one-step assay according to the manufacturer’s protocol.

### 2.13. Tissue Viral Load Assays

Assays to detect and quantify CHIKV-specific amplicons were conducted using the one-step RT-PCR method [24]. The sensitivity of this method was determined using a series of 10-fold dilutions of in vitro transcribed RNA, generated from a cloned cDNA copy of the amplicon (pCH127) as the template. The 127 bp cDNA amplicon was cloned downstream from a T7 promoter sequence in the pMK plasmid vector by GeneArt (Ambion™ ThermoFisher Scientific, UK). An XbaI site was engineered immediately downstream from the 3′ end of the insert to enable linearization prior to in vitro transcription using the MEGAshortscript™ kit (Ambion™ ThermoFisher Scientific). The template copy number in the transcribed RNA was estimated [25] and plotted against the Ct value obtained for each dilution.

### 2.14. IFN Assays

The expression of type I IFN at a range of time points following infection was evaluated in RNA purified from cell culture lysates and tissue homogenates using IFNa2 and IFNb1 “Assays on demand™” (Life Technologies, ThermoFisher Scientific) according to the manufacturer’s instructions. Prior to PCR-amplification, cDNA was prepared from 50 µL of eluted RNA after adding an equal volume of RT master-mix taken from a High-Capacity cDNA Reverse Transcriptase kit (Applied Biosystems, Warrington, UK). For each gene of interest, 16 µL per sample of pre-mix and 4 µL cDNA (test samples) or nuclease-free water (negative controls) were distributed into the appropriate wells of a MicroAmp^®^ Fast Optical 96-well reaction plate (Applied Biosystems, UK). Thermocycling was carried out using an Applied Biosystems 7500 Fast Real-Time PCR System, with the following conditions: 50 °C for 2 min, 95 °C for 10 min, and 40 cycles of 95 °C for 15 s and 60 °C for 1 min. Samples were run in triplicate and the mean average Ct values calculated.

## 3. Results

### 3.1. Growth Characteristics In Vitro

**Figure 2:**
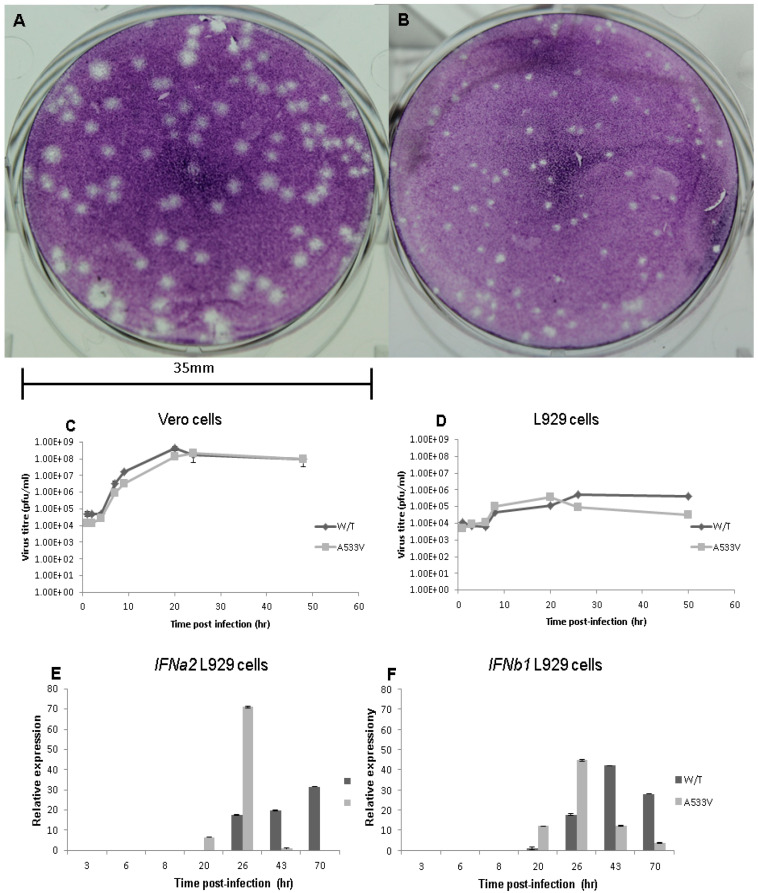
Evaluation of growth characteristics of wild-type and A533V-mutant CHIKV clones in vitro. The plaque morphology in Vero cells infected with wild-type (**A**) and A533V-mutant (**B**) clones are shown. Cell cultures grown to 90–100% confluence were infected at a MOI of 0.01 and incubated in DMEM containing 5% FBS at 37 °C. Supernatants from Vero (**C**) and L929 (**D**) cell lines were removed at the times indicated and virus titres estimated by plaque assay. Error bars denote standard deviation. Total RNA extracted from L929 cell cultures harvested at various periods post-infection was assayed in triplicate by two-step TaqMan qRT-PCR to determine the expression of IFNa2 (**E**) and IFNb1 (**F**) genes.

Both wild-type and A533V-mutant viruses are replicated and induced CPE in Vero cells. Plaques resulting from Vero cell monolayers infected with wild-type virus displayed a larger phenotype with a less regular border than those from the mutated virus (Figure 2A,B). When plaque assays with the two virus types were conducted under identical conditions, the average plaque diameter produced by the wild type was 3.34 mm (n = 50, SD = 0.348993), whereas that produced by the A533V mutant was 1.64 mm (n = 50, SD = 0.161271).

In order to investigate whether or not the observed effect of the A533V mutation on plaque morphology was associated with a defect in replication compared to the wild-type virus, the growth kinetics of wild-type and A533V-mutant CHIKV clones were investigated using Vero and L929 cell lines. Whereas both are susceptible to alphavirus infection, the former cell line differs from the latter by being defective in its ability to secrete type I IFN [26,27]

Both viruses reached a peak titre in the range of 2.6–2.8 × 10^8^ pfu/mL after 20–24 h in Vero cells; however, at the earlier time points for which plaque assays were conducted, the mutant virus grew to lower titres than the wild-type virus (Figure 2C,D). Infection of L929 cells with the A533V-mutant virus resulted in higher viral yields than those observed with wild-type virus at the early time points, reaching a peak at 20 h, but this was followed by a marked reduction in yields. The peak virus titre coincides with the induction of type 1 IFN as indicated by the earliest detection of IFNa2 and IFNb1 (Figure 2E,F) gene transcripts from infected cell supernatants. By contrast, the titre of the wild-type virus remained relatively high at the final time point and the concomitant type 1 IFN gene expression was delayed relative to the A533V-mutant virus.

### 3.2. Effect of the A533V Mutation on Virus Phenotype In Vivo

**Figure 3:**
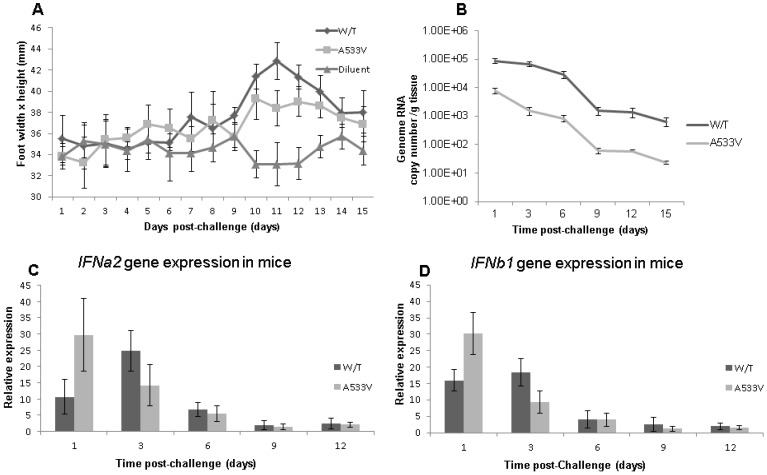
Disease in CHIKV-challenged adult C57BL/6 mice. The extent of foot swelling following inoculation was monitored for 15 days; this was assessed by multiplying the width and height of the foot to provide the approximate area in mm^2^ (**A**). RNA was extracted from inoculated limbs at a range of time points post-inoculation and subjected to assays to provide a measure of viral load and relative expression of type I IFN genes. The qRT-PCR method of Edwards et al. (2007) [24] was used to detect CHIKV RNA and the viral load in terms of approximate genome copy number was estimated from C(t) values (**B**). Expression of *IFNa2* (**C**) and *IFNb1* (**D**) genes was determined using the High-Capacity cDNA Reverse Transcriptase kit (Applied Biosystems, UK) followed by gene-specific PCR assays (“Assays on demand™”, Life Technologies, ThermoFisher Scientific). All assays were performed in triplicate and the mean average C(t) normalised with HPRT. Error bars denote standard deviations.

Mice appeared healthy throughout the course of the experiment with no significant differences in body temperature or fluctuations in weight gain observed in the CHIKV-challenged animals (wild-type and A533V-mutant examples) compared to mock challenge controls. However, significant swelling in the tarsus region in the inoculated limb was seen between days 9 and 13 post-exposure in mice receiving wild-type and A533V-mutant viruses (Figure 3A). Swelling was more severe in the former than in the latter and was absent in group 3 (mock-challenged controls).

### 3.3. Assessment of Tissue Viral Load by qRT-PCR

A decrease in the viral load was observed for successive samples in homogenized leg tissue at each time point from days 1 to 15 post-exposure. However, this was most marked between days 6 and 9 post-exposure, the period immediately prior to the development of foot swelling (Figure 3A,B), and was of a similar duration to the viraemic phase reported for CHIKV isolates with the same mouse model by Gardner et al. (2010) [25]. At each time point, the mean viral genome copy number was a minimum of 1.0 log higher in mice challenged with the wild-type virus than those receiving the A533V-mutant virus.

The relative quantity of transcripts for IFNa2 and IFNb1 genes in total RNA extracted from homogenized mouse leg tissue was determined by qRT-PCR in order to assess the effect of the A533V mutation on type 1 IFN induction (Figure 3C,D). In common with the data obtained in vitro, a greater quantity of each transcript was stimulated more rapidly in the A533V-mutant virus than in the wild-type virus. Expression of both genes was significantly higher on day 1 post-infection in mice receiving the A533V-mutant virus than those receiving the wild-type virus, a state that was reversed by day 3 post-infection.

**Figure 4:**
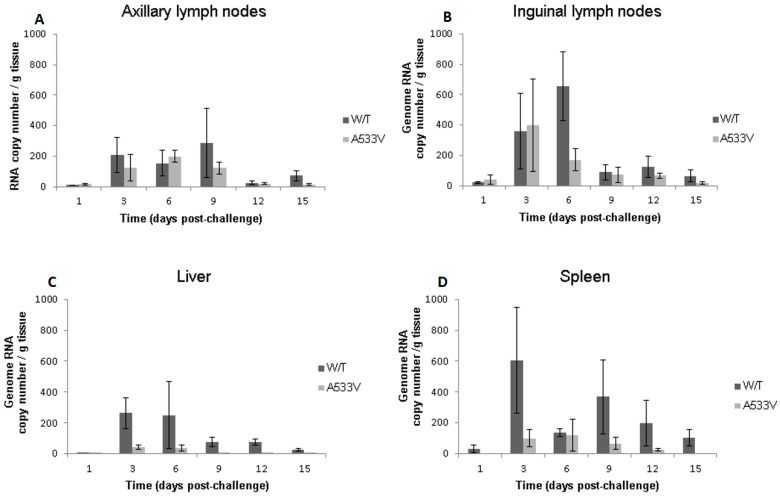
Viral loads in mouse tissues from animals infected subcutaneously with 1 × 10^4^ pfu CHIK-wild-type or A533V-mutant viruses diluted in a total of 40 µL DMEM medium containing 5% FBS. Axillary lymph nodes (**A**), inguinal lymph nodes (**B**), liver (**C**), and spleen (**D**) were assayed for CHIKV RNA by qRT-PCR by Edwards et al. (2007) [24] and the C(t) values were converted into the approximate RNA copy numbers. At each time point indicated, assays were performed in triplicate on tissues from each of three animals and the mean average values shown with error bars denoting standard deviation.

Viral load assays were conducted in tissues from additional sites in order to compare the dissemination of the two viruses (Figure 4A–D). In all cases, the viral loads were higher in tissues obtained from animals infected with the wild-type virus than in those infected with the A533V-mutant virus, indicating an attenuated phenotype in the latter. In lymph node tissues, the virus was detected at all time points with the peak titres being observed at 3 to 9 days (axillary) or 3 to 6 days post-challenge (inguinal). Evidence of dissemination to the liver and spleen differed between wild-type and A533V-mutant viruses. In the liver and spleen, peak wild-type virus loads were detected at 3 days post-exposure, thereafter gradually declining, whereas levels of the A533V-mutant virus were near or below the limit of detection at all time points.

### 3.4. Histopathology Findings

Tissues from the un-inoculated control group were normal for all the parameters examined. In the two challenge groups and the diluent-only group, significant changes were confined to connective tissues (Table 3), muscle (Table 4), and to a lesser extent, skin in the tarsal region.

In fibrovascular tissue from mice receiving the wild-type virus, a minimal, predominately mononuclear, inflammatory cell infiltrate with scattered polymorphonuclear leucocytes (PMNs) was observed in 2/3 animals on day 1 post-challenge (pc). Changes increased in severity, with 3/3 mice exhibiting marked signs of inflammation on days 6 and 9 pc and subsequently decreased by days 12 and 15 pc. In mice receiving the A533V-mutant virus, a similar inflammatory cell infiltrate was observed. This was assessed as normal, minimal, or mild on days 1–6 pc, minimal to moderate by day 9 pc, and normal, minimal, or mild on days 12 to 15 pc. Minimal changes were observed in a minority (2/18) of mice in the diluent-inoculated control group (one each on days 1 and 15 pc).

Myocyte degeneration was seen in the majority of animals at each time point. Tissues were either normal or showed minimal changes at days 1 and 3 pc from mice in all groups, except for a single animal receiving the wild-type virus, in which mild changes were reported. Thereafter, in the wild-type virus-challenged group, 3/12 had mild changes and 9/12 had moderate or marked changes. In the A533V-mutant-challenged mice, 2/12 animals had moderate changes on day 9 (pc), whilst in the remainder, changes were rated as normal, minimal, or mild at the remaining time points. From days 3 to 15 pc, inflammatory cell infiltrate ranging from minimal to marked was reported in 13/15 wild-type virus-challenged mice and from minimum to moderate in 5/15 of those receiving the A533V-mutant virus. Minimal changes were observed in individuals from all time points from the diluent-inoculated control group (9/18).

In the dermis, changes comprised a mainly mononuclear inflammatory cell infiltrate with scattered polymorphonuclear leukocytes (PMNs). Changes ranging from minimal to moderate were reported in 12/18 animals receiving the wild-type virus and minimal in 2/18 animals receiving the A533V-mutant virus.

**Figure 5:**
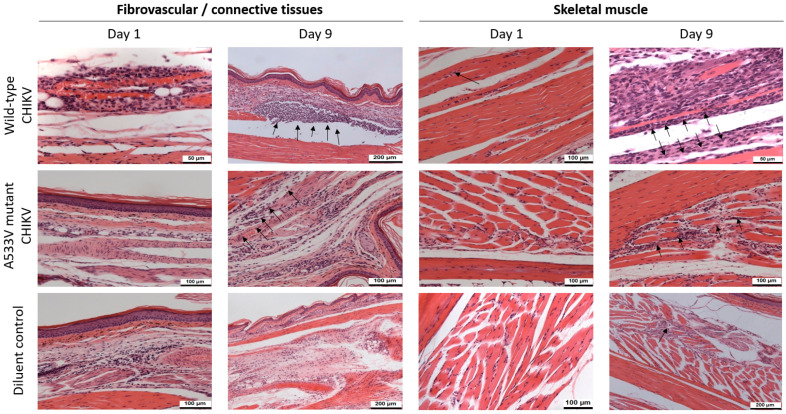
Adult C57BL/6 mice were challenged intradermally with 1 × 10^4^ pfu of either wild-type or A533V-mutant CHIKV diluted in 40 µL cell culture medium into the distal hind limb. A diluent control group was inoculated in a similar manner with culture medium only. Groups of three mice were euthanized at days 1, 3, 6, 9, 12, and 15 post-challenge and H&E stained sections prepared and assessed for indicators of pathology. Representative images of subcutaneous connective and skeletal muscle tissue obtained from the inoculated tarsus region at days 1 and 9 post-challenge are shown. Minimal changes in connective tissue (inflammatory cell infiltrates) were observed from day 1 post-inoculation (arrow); in animals challenged with both wild-type and A533V-mutant viruses, the severity of these changes progressively increased until day 9, thereafter declining (not shown). Similarly, changes associated with degeneration and necrosis/loss of muscle fibres and a variable inflammatory infiltrate, were most severe at day 9 post-challenge. The changes observed in both tissue types were more severe in animals challenged with the wild-type virus than A533V-mutant virus. Minimal changes were observed in connective tissue from two animals and in muscle fibres from eight.

Changes suggestive of inflammation in the synovial membranes were observed only in mice receiving the wild-type virus. These were rated as moderate on day 6, mild in 1/3 animals on day 9, and minimal in 2/3 animals on day 15 pc. Figure 5 displays H&E staining images for fibrovascular/connective tissues and skeletal muscle tissue at days 1 and 9 post-challenge with either the wild-type virus, A553V-mutant virus, or diluent control.

## 4. Discussion

The pathogenesis of alphavirus-induced disease appears to be determined by several virus-specific factors working in concert and includes mechanisms enabling them to counteract the effects of the innate and adaptive responses by the host immune system. It is well recognised that the type I IFN pathway is crucial in controlling acute CHIK disease [11,12,13] and that members of the Alphavirus genus have evolved mechanisms enabling them to interfere with the effects of type I IFN induction.

In the present study, it is shown that the introduction of a single amino acid substitution to the nsP1 (A533V) in an otherwise wild-type CHIKV results in a viable virus, albeit with altered growth kinetics in vitro and reduced pathogenicity in the adult C57BL/6 mouse model.

Although both the wild-type and A533V-mutant CHIKV clones reached similar peak titres in infected Vero cell cultures at approximately 20–24 h post-infection, the latter exhibited lower titres at earlier time points. In addition, the A533V-mutant virus displayed a smaller plaque phenotype than the wild-type virus, a finding reported in both SINV and RRV following the introduction of mutations at this site [16,22].

Peak titres of both viruses were significantly lower in L929 cells than those observed with Vero cells (a cell line unable to mount a type I IFN response); however, the decline in the wild-type clone titre was significantly less marked than that observed with the A533V-mutant clone. Evidence to suggest that this effect was due to the ability of L929 cells to mount a type1 IFN response was provided by the observation that higher relative quantities of the transcripts of IFNa2 and IFNb1 genes were detected at an earlier post-infection time point in the A533V-mutant group than in the wild-type virus group.

Changes associated with the two viral infections were observed in the challenged mice in the present study. Foot swelling was observed only in the animals receiving viral inocula and was more marked in animals receiving the wild-type virus than in those receiving the A533V-mutant virus.

Interestingly, the appearance of foot swelling near the site of inoculation in the present study (9–13 days post-challenge) occurred significantly later than that observed by Gardner et al. [23] who reported this feature at 7–9 days post-challenge. It is possible that differences in the cell line used for the preparation of virus inoculum may have contributed to this effect. Although Vero cells are unable to express type I IFN genes, the induction of IFN-stimulated genes by IFN-independent pathways [28,29] may have rendered the inoculum used in the present study less infectious than that prepared in the insect cell line C6/36 by Gardner et al. [23].

In addition, the timing of the foot swelling was correlated, approximately, with histological changes observed in the lower limb tissue. These were most marked in tarsal skeletal muscle and connective tissues, increasing in severity from day 1 to day 9 after infection and declining thereafter. Although minimal histological changes were observed in some animals in the diluent control group, the absence of swelling in the feet of these animals indicate that the more severe changes in the inoculated group were associated with the viral challenge. The findings indicative of localized arthritic pathology are essentially similar to those of Gardner et al. [23].

Viraemia (indicated by CHIKV RNA in muscle tissue) was detected in mice infected with wild-type CHIKV on days 1, 3, and 6 post-challenge, whereas markedly lower levels of shorter duration (on days 1 and 3 only) were detected in mice receiving the A533V-mutant virus.

The viral load detected at each time point was consistently higher in tissues derived from animals infected with the wild-type than those infected with the A533V-mutant virus, indicating that the modified genotype has an attenuating effect. Furthermore, viral RNA was detected in tissue extracted from the inoculated limbs of animals receiving the wild-type virus at all time points, whereas the A533V-mutant viral RNA was not detected beyond day 3 post-challenge in animals receiving the mutant virus. Viral RNA was detected in liver homogenates from day 3 post-challenge in mice challenged with both virus types, declining to undetectable levels by day 12 for those receiving the wild-type virus and day 9 for those receiving the A533V-mutant virus. In contrast, evidence of virus presence in the axial and inguinal lymph nodes and in the spleen persisted until the end of the study. These findings differ with those of Gardner et al., who reported the detection of the virus in the spleen, inguinal lymph nodes, and liver for shorter periods post-challenge (until day 5 in lymph nodes and spleen and day 3 in liver tissues) [25]. This can be explained by the different methods used for virus detection; the qRT-PCR assay used in the present study detects genome RNA copy numbers, whereas the endpoint dilution assay used by the authors of the former study detects viable virus particles.

Taken together, these results show that the introduction of the nsP1 A533V mutation to an otherwise wild-type clone results in progeny with altered growth characteristics from the parental virus and induces type 1 IFN at an earlier stage in the infectious process. Furthermore, in vivo studies show that virus containing this mutation is significantly attenuated in the C57BL/6 mouse model. Given that a similar determinant has previously been reported in SINV and RRV [22] and that Old World alphaviruses exhibit relatively similar conserved motifs at this site [1], it may be reasonable to speculate that other members of this group share this phenotype. These results provide further evidence that this virulence determinant is widespread amongst Old World alphaviruses and thus a potential target for the development of future antiviral agents.

## Figures and Tables

**Table 1:** Oligonucleotide primers used to produce CHIKV genome amplicons used for the construction of the CHIKV cDNA clone.

Primer	Sequence	Genome Location
CH1F	ATGGCTGCGTGAGACACAC	1–19
CH3090R	GTATCGAAGGTCATTTGGTGA	3071–3090
CH2971F	GTGGATAAAGACGCTGCAGAAC	2971–2992
CH5864R	GCGACTGATACCTGCTTCTGTT	5843–5864
CH5306F	ACTGTGACATGTGACGAGAGAG	5306–5327
CH8273R	GCTCCTTCATTAGCTCCTCCTAA	8251–8273
CH7924F	GAAGGAAGGTAACAGGTTACG	7924–7945
CH9585R	GGCTGTACCGTTTGTAGATAACT	9563–9585
CH9441F	GGAGAAGAACCAAACTATCAAGAAG	9441–9466
CH1811R	GAAATATTAAAAACCAAAATAACATCTCCTA	11,783–11,811
CH9384F	CCAAGTCATCATGCTACTGTATCC	9384–9407
CHIK3′(A15)R	TTTTTTTTTTTTTTTGAAATATTAAAAACAAAATAACATCTCC	11784-A(15)
CHIK3′(A40)ApaI	GGGCCCTTTTTTTTTTTTTTTTTTTTTTTTTTTTTTTTTTTTTTTTGA	11810-A(40)ApaI

**Table 2:** Amplicons used to construct cDNA sub-clones of the CHIKV strain SL-R-233 (2006) genome.

Sub-Clone	Primers	Amplicon Size (bp)	Genome Location/Gene
S1	CH1F/CH3090	3090	5′UTR/nsP1/nsP2
S2	CH2971F/CH5864R	2893	nsP2/nsP3/nsP4
S3	CH5306F/CH8273R	2966	nsP3/nsP4/C
S4	CH7924F/CH9585R	1665	C/E3/E2
S5	CH9441F/11811R	2327	E2/6K/E1/3′UTR
T1	CH9384F/CH(A15)Term	2427	E2/6K/E1/3’UTR/A(15)
T2	CH9384F/CH(A40)ApaI	2452	E2/6K/E1/3’UTR/A(40)

**Table 3:** Histopathological changes in fibrovascular connective tissues were assessed in haematoxylin and eosin-stained tissue sections from the tarsus region of C57BL/6 mice challenged with 40 µL of cell culture medium containing 1 × 10^4^ pfu wild-type CHIKV, 1 × 10^4^ pfu A533V-mutant CHIKV, or diluent control (DMEM). Three mice from each group were euthanized on the days indicated and samples visualised by light microscopy. Samples were assessed and allotted to one of the five categories shown to indicate the extent of histological change observed.

Day	Group	Severity of Histological Changes: Connective Tissue				
		Normal	Minimal	Mild	Moderate	Marked
**1**	Wild type	1	2	-	-	-
	A533V mutant	2	1	-	-	-
	Control	2	1	-	-	-
**3**	Wild type	-	-	2	-	1
	A533V mutant	3	-	-	-	-
	Control	2	1	-	-	-
**6**	Wild type	-	-	-	-	3
	A533V mutant		1	2	-	-
	Control	3	-	-	-	-
**9**	Wild type	-	-	-	-	3
	A533V mutant	-	1	1	1	-
	Control	3	-	-	-	-
**12**	Wild type	-	-	1	2	-
	A533V mutant	1	1	1	-	-
	Control	3	-	-	-	-
**15**	Wild type	-	-	2	1	-
	A533V mutant	2	1	-	-	-
	Control	3	-	-	-	-

**Table 4:** Histopathological changes in skeletal muscle specimens were assessed in haematoxylin and eosin-stained tissue sections from the tarsus region of C57BL/6 mice challenged with 40 µL of cell culture medium containing 1 × 10^4^ pfu wild-type CHIKV, 1 × 10^4^ pfu A533V mutant CHIKV, or diluent control (DMEM). Three mice from each group were euthanized on the days indicated and samples visualised by light microscopy. Samples were assessed and allotted to one of the five categories shown to indicate the extent of histological change in terms of myocyte degeneration. Inflammatory cell infiltrate (where observed) is indicated in brackets.

Day	Group	Severity of Histological Changes: Skeletal Muscle				
		Normal	Minimal	Mild	Moderate	Marked
**1**	Wild type	-	3	-	-	-
	A533V mutant	2	1	-	-	-
	Control	2	1	-	-	-
**3**	Wild type/T	1	1 (1)	1	-	-
	A533V mutant	1	2	-	-	-
	Control	1	2	-	-	-
**6**	Wild type	-	-	1 (1)	2	-
	A533V mutant	-	1	2	-	-
	Control	2	1	-	-	-
**9**	Wild type	-	-	-	1	2 (3)
	A533V mutant	-	-	1	2 (1)	-
	Control	1	2	-	-	-
**12**	Wild type	-	(1)	2 (1)	1 (1)	-
	A533V mutant	-	2	1 (1)	-	-
	Control	1	2	-	-	-
**15**	Wild type	-	-	(3)	1	2
	A533V mutant	-	2 (3)	1	-	-
	Control	3	-	-	-	-

## Data Availability

The data presented in this study are available on request from the corresponding authors.
